# *Sclerotinia sclerotiorum* Thioredoxin Reductase Is Required for Oxidative Stress Tolerance, Virulence, and Sclerotial Development

**DOI:** 10.3389/fmicb.2019.00233

**Published:** 2019-02-14

**Authors:** Jinyi Zhang, Yabo Wang, Jiao Du, Zhiqiang Huang, Anfei Fang, Yuheng Yang, Chaowei Bi, Ling Qing, Yang Yu

**Affiliations:** College of Plant Protection, Southwest University, Chongqing, China

**Keywords:** *Sclerotinia sclerotiorum*, thioredoxin reductase, oxidative stress, sclerotia, virulence, gene silencing

## Abstract

*Sclerotinia sclerotiorum* is a destructive ascomycete plant pathogen with worldwide distribution. Extensive research on different aspects of this pathogen’s capability to cause disease will help to uncover clues about new ways to safely control Sclerotinia diseases. The thioredoxin (Trx) system consists of Trx and thioredoxin reductase (TrxR), which play critical roles in maintenance of cellular redox homeostasis. In this study, we functionally characterized a gene encoding a TrxR (*SsTrr1*) in *S. sclerotiorum*. The amino acids of SsTrr1 exhibited high similarity with reported TrxRs in plant pathogens and targeted silencing of *SsTrr1* lead to a decrease in TrxR activities of mycelium. *SsTrr1* showed high expression levels during hyphae growth, and the levels decreased at the different stages of sclerotial development. *SsTrr1* gene-silenced strains produced a smaller number of larger sclerotia on potato dextrose agar medium. The observations were consistent with the inhibitory effects on sclerotial development by the TrxR inhibitor, anrunofin. The expression of *SsTrr1* showed a dramatic increase under the oxidative stress and the hyphal growth of gene-silenced strains showed more sensitivity to H_2_O_2_. *SsTrr1* gene-silenced strains also showed impaired virulence in different hosts. Taken together, our results suggest that *SsTrr1* encodes a TrxR that is of great important for oxidative stress tolerance, virulence, and sclerotial development of *S. sclerotiorum*.

## Introduction

*Sclerotinia sclerotiorum* is an ascomycete plant pathogen with a worldwide distribution (Bolton et al., 2006). This fungus infects more than 400 known plants and is the causal agent of stem rot in oilseed rape. *S. sclerotiorum* produces sclerotia, which are hard, asexual, resting structures. As melanized hyphal aggregates, sclerotia can survive for years in soil and play an important role in the disease cycle (Bolton et al., 2006; Erental et al., 2008). Sclerotia may germinate carpogenically to produce millions of airborne ascospores, which are the primary sources of inocula in most Sclerotinia diseases. Under certain conditions, sclerotia also germinate myceliogenically to produce hyphae, which can directly infect the hosts’ stem or leaves (Schwartz and Steadman, 1978; Bardin and Huang, 2001).

Reactive oxygen species (ROS), including superoxide anion ($O_{2}^{-}$), hydrogen peroxide (H_2_O_2_), and the hydroxyl radical (⋅OH) play important roles as secondary messengers in many intracellular signaling pathways (Mittler et al., 2011; Ray et al., 2012). However, high ROS concentrations can lead to DNA damage, protein inactivation and fragmentation, and lipid peroxidation (Aguirre et al., 2006). In plant–microbe interactions, ROS works as part of a defense mechanism and is a characteristic feature of the hypersensitive response (HR) (Lamb and Dixon, 1997). To detoxify ROS efficiently, cells usually use complex antioxidant responses, which mainly include superoxide dismutases, catalases, peroxidases, glutathione peroxidases, peroxiredoxins, and thioredoxins (Trxs) (Pomposiello et al., 2001; Aguirre et al., 2006).

The Trx system is ubiquitous from eukaryotes to archaea and plays a basic role in the maintenance of the redox environment in cells (Arnér and Holmgren, 2000). The Trx system is composed of Trx, thioredoxin reductase (TrxR), and nicotinamide adenine dinucleotide phosphate (NADPH) (Holmgren, 1989). Trx contains a dithiol/disulfide active site (CGPC) and works as a major cellular disulfide reductase. Using NADPH as an electron donor, TrxR catalyzes the reduction of the active disulfide site in oxidized TrxR, Trx-S_2_, to a dithiol in reduced TrxR, Trx-(SH)_2_ (Arnér and Holmgren, 2000). Reduced Trx directly reduces the disulfide in target proteins, and this process is required for several intracellular processes (Thön et al., 2007). In addition to Trx, TrxR also has other substrates, such as the glutaredoxin-like protein, NrdH, in *Escherichia coli* (Jordan et al., 1997).

TrxR are homodimeric flavoenzymes that belong to a larger family of pyridine nucleotide-disulfide oxidoreductases. They contain an active redox disulfide and binding sites for flavin adenine dinucleotide (FAD) and NADPH in each subunit (Thön et al., 2007). TrxR can be divided into two classes according to the molecular weight (Ghisla and Massey, 1989). High molecular weight TrxR is present in higher eukaryotes and has a molecular weight of 55–58 kDa, while low molecular weight TrxR (homodimers of 35–36 kDa subunits) is present in prokaryotes, archaea, plants, and fungi (Thön et al., 2007).

Recently, some fungal genes that encode TrxR have been cloned and functionally analyzed. Two TrxRs (Trr1 and 2) that have cytoplasmic and mitochondrial locations, respectively, were characterized in *Saccharomyces cerevisiae* (Pearson and Merrill, 1998; Pedrajas et al., 1999). An *S. cerevisiae* strain without *trr2* showed more sensitivity to H_2_O_2_ (Pedrajas et al., 1999). *Beauveria bassiana*, a filamentous fungal insect pathogen, also contains two TrxR genes that play distinct roles in the redox system and host infection (Zhang et al., 2016). Some evidence has shown that the TrxRs are required for pathogenic activity in fungal plant pathogens. A loss of TrxR in *Magnaporthe oryzae* resulted in strains that failed to produce spreading necrotic lesions on the leaf surface (Fernandez and Wilson, 2014). The targeted deletion of TrxR in *Alternaria alternata* led to strains that were defective in H_2_O_2_ detoxification and induced smaller lesions on citrus leaves (Ma et al., 2018). In *Botrytis cinerea*, a fungus closely related to *S. sclerotiorum*, deletion of the TrxR-encoding gene, *trr1*, impaired fungal virulence and antioxidant capabilities (Viefhues et al., 2014). However, the role of TrxR in the development and pathogenicity of *S. sclerotiorum* is still unclear.

In this study, a gene encoding a putative TrxR has been identified, and its function in the sclerotial development and pathogenicity of *S. sclerotiorum* were characterized. The findings could help to advance our understanding of the role of TrxR in fungal plant pathogens and the molecular mechanisms that are involved in the sclerotial development and pathogenicity of *S. sclerotiorum*.

## Materials and Methods

### Fungal Strains and Culture Conditions

The *S. sclerotiorum* isolate “1980” (Godoy et al., 1990) was used as the wild-type strain in this study. Strains were routinely cultured on potato dextrose agar (PDA) (Difco Laboratories, Detroit) at 20°C. Transformants were cultured on PDA supplemented with hygromycin B at 100 μg/mL (Calbiochem, Riverside, CA, United States). The effects of auranofin (MedChem Express, Princeton, NJ, United States) on hyphae growth and sclerotial development were by adding a range of concentrations of auranofin (0–62.5 μM) to the PDA medium.

### Vector Construction and Transform

An *SsTrr1* gene-silencing vector was constructed based on plasmid pCIT (Yu et al., 2012). The primer pairs SiTrr1ClaI (CGCATCGATTCAGCTCGCAGACTCGGTCT)/SiTrr1EcoRV (CGCGATATCTCGTTCCGGGCTTGGTTAC) and SiTrr1 BamHI (CGCGGATCCTCAGCTCGCAGACTCGGTCT)/SiTrr1 PstI (CGCCTGCAGTCGTTCCGGGCTTGGTTAC) were designed according to *SsTrr1* cDNA sequences and then used to amplify the sense and antisense fragments of *SsTrr1*, respectively. The sense and antisense fragments were successfully inserted into the corresponding multiple cloning sites of the pCIT vector. A hygromycin resistance gene was then inserted into the *Xba* I site of the newly constructed vector to create the *SsTrr1* RNA silencing vector pSiTrr1. The vector was then linearized with *Xho* I and used to transform the wild-type protoplasts of *S. sclerotiorum* according to the method used by Rollins (2003).

### Nucleic Acid Manipulation and Real-Time RT-PCR

To assay the expression levels of *SsTrr1* transcripts in different stages of sclerotial development of *S. sclerotiorum*, the wild-type strain was cultured on cellophane over PDA, and mycelia were harvested at 2 days post-inoculation (dpi) (hyphae), 3 dpi (initial sclerotia), 5 dpi (developing sclerotia), and 8 dpi (mature sclerotia). The mature sclerotia were cultured on the surface of moist sand at 16°C and collected once the stipe initials appeared. To evaluate the expression levels of *SsTrr1* transcripts in different transforms containing pSiTrr1, the wild-type strains and the transforms were cultured on PDA for 3 days. To analyze the expression levels of *SsTrr1* under oxidative stress conditions, the wild-type strain was cultured on PDB for 1 day, and then the culture was treated with 10 mM H_2_O_2_ for 1 day. The RNA products in different samples were extracted with a Trizol reagent (TianGen, Dalian, China). First-strand cDNA synthesis was performed using a ReventAid^TM^ First Strand cDNA Synthesis Kit (MBI Fermentas, Flamborough, ON, Canada). The relative expression levels of *SsTrr1* were obtained with real-time reverse-transcriptase polymerase chain reaction (RT-PCR) using a CFX96^TM^ Real-time System (BioRad, Hercules, CA, United States). Real-time RT-PCR assays were performed according to Yu et al. (2012) with primer pair RT-SsTrr1fp (AGAATTTCCCTGGTTTCCCTAA)/RT-SsTrr1rp (GTGTTCTGTCTTGTCATCCCATT), which was designed based on the cDNA of *SsTrr1*. The β-tubulin gene *tub1* (SS1G_04652) was used as an internal control and amplified with the primer pair RT-tubfp (GTGAGGCTGAGGGCTGTGA)/RT-tubrp (CCTTTGGCGAT GGGACG).

### Pathogenicity Assay

Pathogenicity assays were conducted on *Arabidopsis thaliana* col-0 and *Nicotina benthamiana* according to Yu et al. (2017) with slight modification. All plants were grown in a greenhouse at 25°C under a 16-h light/8-h dark cycle. The plants or leaves were inoculated with mycelium agar plugs (6 mm in diameter) obtained from the edges of the growth colony of the wild-type strain and *SsTrr1* gene-silenced strains. Photographs were taken at 24 and 48 hpi for *N. benthamiana* and *A. thaliana*, respectively. The experiment was repeated at least three times, and each strain was evaluated with at least five plants with three leaves (15 leaves total).

### Oxidative Stress Treatment

In order to test the effects of oxidative stress on hyphal growth, the wild-type strain and *SsTrr1* gene-silenced strains were cultured on PDA medium and PDA with H_2_O_2_ (5, 10 mM). The colony diameters were then measured at 36 h to determine the inhibition of hyphal growth. Each experiment was repeated at least three times.

### Thioredoxin Reductase Activity Measurements

The wild-type strain and *SsTrr1* gene-silenced strains were cultured on PDA medium for 3 days. The total proteins for each strain were then extracted and used for the measurement of TrxR activities using a 5,5′-dithiobis-(2-nitrobenzoic acid) (DTNB) assay with assay kits (Solarbio, Beijing, China). Absorbance values were monitored at 412 nm. TrxR activity levels were expressed as U/g mycelium. The unit U/g refers to the amount of TrxR in a 1-g sample that catalytic reduction of 1 μmol DTNB per minute.

## Results

### Auranofin Inhibits the Hyphal Growth and Sclerotial Development of *S. sclerotiorum*

**FIGURE 1:**
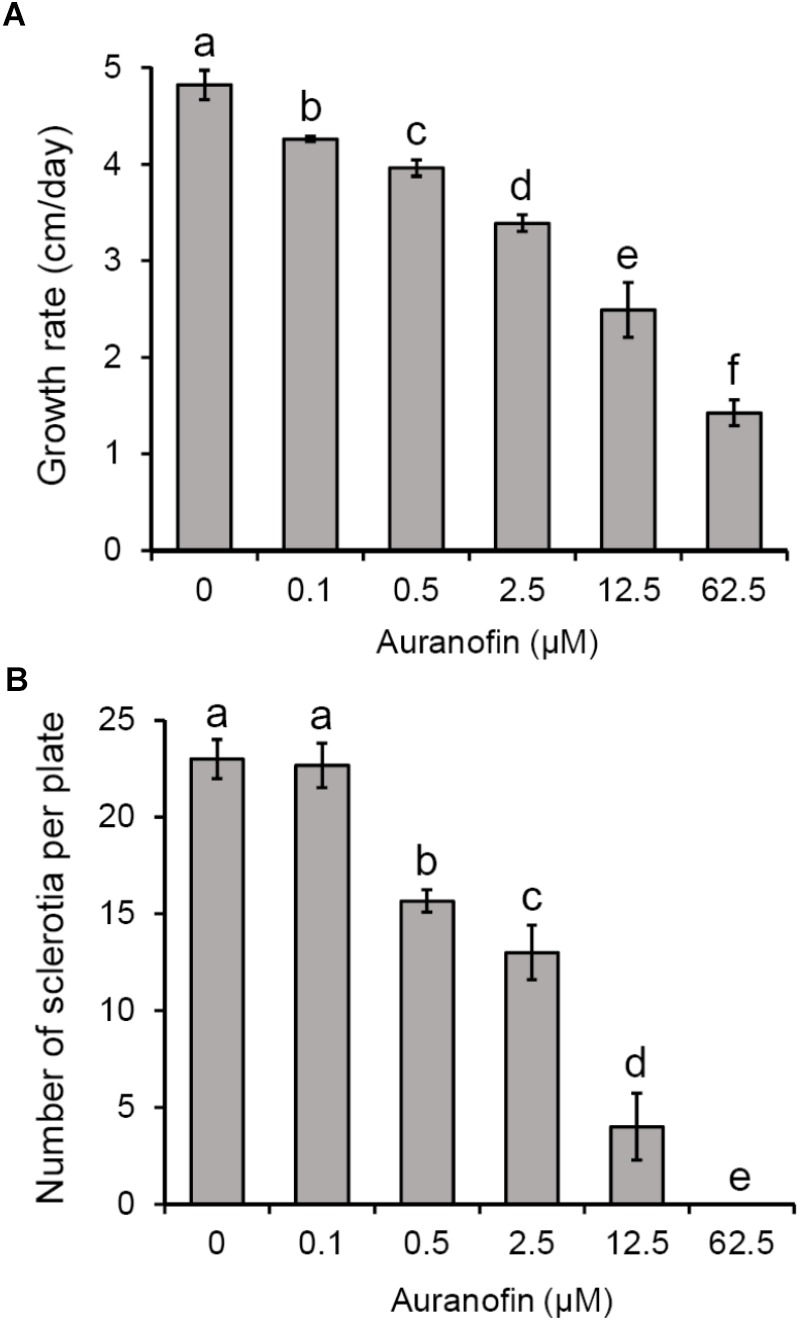
Effects of auranofin on *S. sclerotiorum* colony growth and sclerotial formation. **(A)** Colony growth of *S. sclerotiorum* on PDA medium with auranofin at concentrations of 0–62.5 μM. **(B)** Number of sclerotia on PDA medium with auranofin. Bars indicate standard deviation. Statistical significance was determined by one-way ANOVA and *post hoc* Tukey’s tests. Different letters in the graph indicate statistical differences (*P* < 0.05).

In order to determine whether TrxR activity is involved in the development of *S. sclerotiorum*, the effects of the TrxR inhibitor auranofin on the hyphal growth and sclerotial formation were examined. The results showed that hyphal elongation was inhibited in the presence of auranofin in PDA medium (50% effective concentration [EC_50_] = 4.4 μM) (Figure 1A). The number of sclerotia was negatively correlated with increasing concentrations of auranofin (Figure 1B), suggesting that auranofin inhibited *S. sclerotiorum* sclerotial formation. These results reveal the inhibitory effect of auranofin on *S. sclerotiorum* and support the possibility that TrxR is required for the hyphal growth and sclerotial development of this fungus.

### Characterization of Thioredoxin Reductase Gene in *S. sclerotiorum*

**FIGURE 2:**
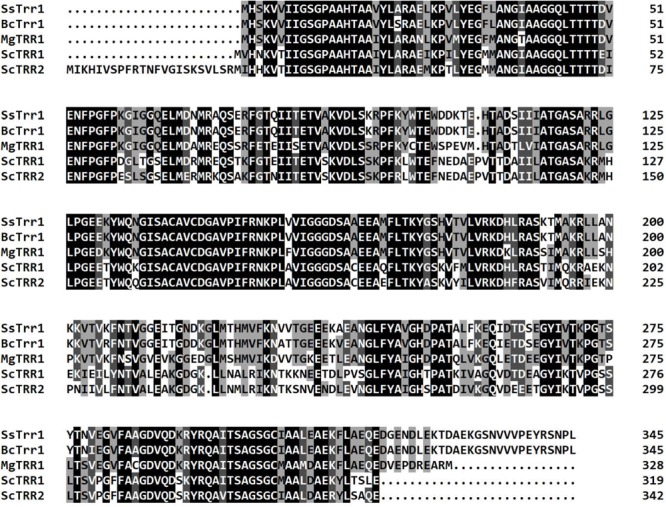
Clustal X alignment of the amino acid sequence of *SsTrr1* with several reported TrxRs of different fungi, including *M. oryzae* TRR1 (EHA54395.1), *B. cinerea* BcTrr1 (XP_001560033.1), *S. cerevisiae* Trr1 (KZV12592.1), and Trr2 (KZV12592.1). Shading indicates sequence similarities of 100 (dark), 75 (medium), and 50% (light). Numbers mean the amino acid of the predicted polypeptide.

**FIGURE 3:**
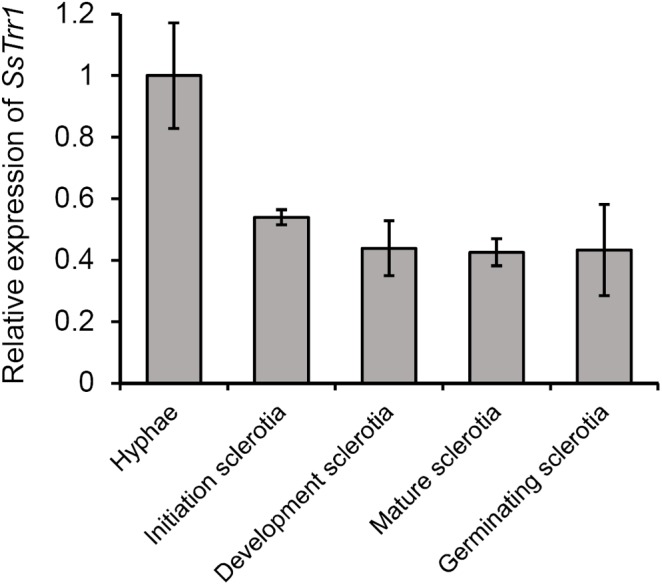
*SsTrr1* transcript in different sclerotial development stages as determined by real-time reverse transcription-polymerase chain reaction (RT-PCR). The quantity of *SsTrr1* cDNA was normalized to that of *tub1* cDNA in extracts from each developmental stage. The abundance of cDNA in the mycelium sample was set as unity. Bars indicate standard error.

In order to explore the roles of TrxR in the development and pathogenicity of *S. sclerotiorum*, one candidate TrxR-encoded gene (SS1G_05899) was identified using the genome sequence of *S. sclerotiorum* (Amselem et al., 2011). The gene comprises five exons encoding a 346-amino-acid polypeptide, which contain a TRX reductase domain at amino acid positions K^4^ to L^306^ (*E*-value = 2.18*e-*145) according to a Conserved Domain Database (CDD) analysis (Marchler-Bauer et al., 2017). The sequence alignment demonstrated that the protein exhibited greater similarity with *B. cinerea* BcTrr1 (97% identity and 100% query coverage) (Viefhues et al., 2014), *M. grisea* MgTRR1 (82% identity and 96% query coverage) (Fernandez and Wilson, 2014), *S. cerevisiae* ScTRR1 (67% identity and 94% query coverage), and ScTRR2 (66% identity and 99% query coverage) (Pearson and Merrill, 1998; Pedrajas et al., 1999) (Figure 2). Thus, the gene was named *SsTrr1* (*S. sclerotiorum* TrxR 1). To provide initial insight into the role of TrxR in fungal development, a real-time RT-PCR analysis was used to measure the abundance of *SsTrr1* mRNA in different growth stages of *S. sclerotiorum*. As shown in Figure 3, *SsTrr1* exhibited constitutive expression at different sclerotia development stages. However, the expression levels in the hyphae were twice that seen during the sclerotial development.

### Functional Analysis of *SsTrr1* in *S. sclerotiorum*

**FIGURE 4:**
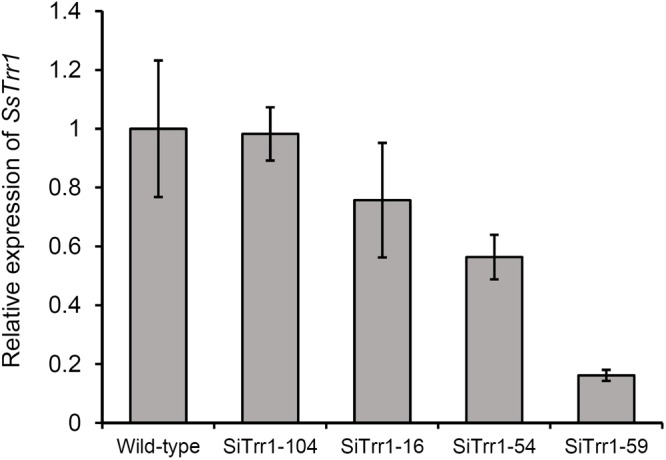
*SsTrr1* transcript in isolates containing pSiTrr1 and in the wild-type strain as determined by real-time RT-PCR. The quantity of *SsTrr1* cDNA was normalized to that of *tub1* cDNA in extracts from each strain. The abundance of cDNA from wild-type strain samples was assigned a value of 1. Bars indicate standard error.

**FIGURE 5:**
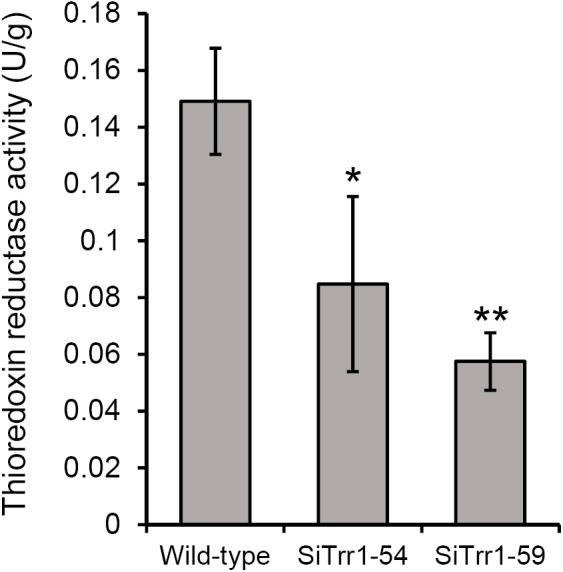
Total TrxR activity quantified in the extracts of 3-day-old PDA cultures of *SsTrr1* gene-silenced strains. Bars indicate standard deviation. Asterisks denote significant differences (one-way ANOVA). ^∗^*P* < 0.05; ^∗∗^*P* < 0.01.

The gene-silenced vector pSiTrr1 was constructed as descripted in the section “Materials and Methods” in order to functionally analyze *SsTrr1* in *S. sclerotiorum*. The vector was used to transform the wild-type strain 1980 as described by Rollins (2003), and several transforms were obtained. *SsTrr1* expression levels in several different randomly selected transformants were determined with real-time RT-PCR, and SiTrr1-54 and SiTrr1-59 showed a dramatic decrease in the abundances of *SsTrr1* mRNA (Figure 4). Thus, these two strains were chosen for further study. The wild-type strain and *SsTrr1* gene-silenced strains were cultured for 3 days, and the TrxR activity in the mycelium was then determined using a DTNB assay. The results showed that the activities of TrxR in SiTrr1-54 and SiTrr1-59 were significantly decreased in comparison with that in the wild-type strain (Figure 5). This suggests that the inhibition of the *SsTrr1* expression levels leads to reduced TrxR activities in *S. sclerotiorum*.

### *SsTrr1* in Relation to Sclerotia Development

**FIGURE 6:**
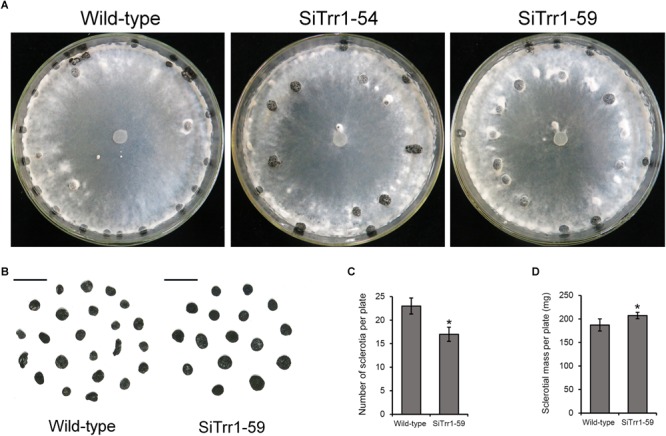
Colony morphology and sclerotial formation of *SsTrr1* gene-silenced strains on PDA medium. **(A)** Colony morphology. **(B)** Sclerotia phenotype. Scale bar = 1 cm. **(C)** Number of sclerotia produced in 90 mm petri plates. **(D)** Sclerotia mass per plate. Bars indicate standard deviation. Asterisks denote significant differences (one-way ANOVA). ^∗^*P* < 0.05.

When cultured on PDA plates, the two *SsTrr1* gene-silenced strains showed similar morphology of the hyphal branch to that of the wild-type strain (data not shown). However, the *SsTrr1* gene-silenced strains produced less of the slightly bigger sclerotia that formed in a random manner in the plates (Figure 6A,B). The average numbers and dry weights of the sclerotia produced by SiTrr1-59 per 9-cm plate were approximately 74 and 110% of those produced by the wild-type strain, respectively (Figure 6C,D). The results were consistent with previous findings of sclerotial formation being inhibited by auranofin, suggesting that *SsTrr1* is related to sclerotial development in *S. sclerotiorum*.

### *SsTrr1* Related to Pathogenicity

**FIGURE 7:**
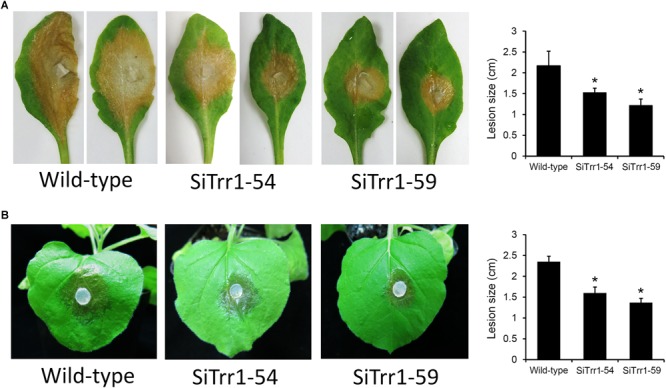
Pathogenicity analysis of *SsTrr1* gene-silenced strains. Detached leaves of *A. thaliana* **(A)** and intact *N. benthamiana* plants **(B)** were inoculated with PDA plugs colonized with the wild-type strain, SiTrr1-54 and SiTrr1-59. Lesion size were measured at 24 and 48 hpi for *N. benthamiana* and *A. thaliana*, respectively. Error bars indicate standard deviation. Statistical significance is indicated in the graph (one-way ANOVA). ^∗^*P* < 0.05.

In order to determine the effects of *SsTrr1* silencing on the pathogenicity of *S. sclerotiorum*, detached *A. thaliana* leaves were inoculated with mycelium plugs of *SsTrr1* gene-silenced strains. As demonstrated in Figure 7A, SiTrr1-54 and SiTrr1-59 led to small lesions on the *A. thaliana* leaves compared to the wild-type strain. The pathogenicity of the two gene-silenced strains was also tested on intact *N. benthamiana* plants, and smaller lesions were observed on the leaves (Figure 7B). These results indicate that *SsTrr1* is required for the full virulence of *S. sclerotiorum*.

### *SsTrr1* in Relation to Oxidative Stress Tolerance

**FIGURE 8:**
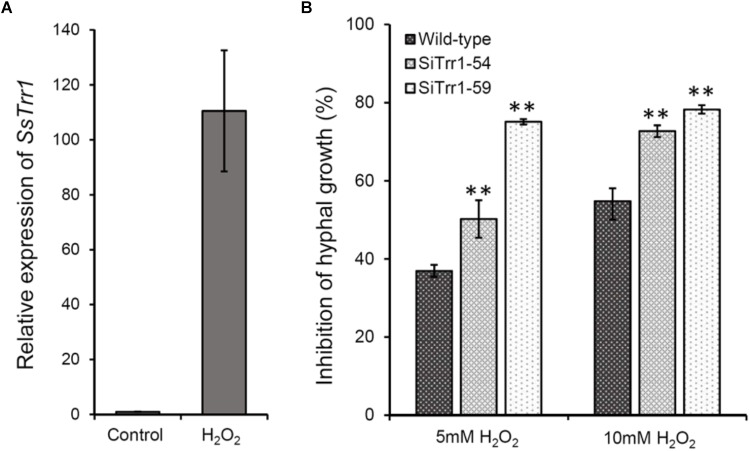
Functional analysis of *SsTrr1* in oxidative stress response. **(A)** *SsTrr1* transcript in hyphae treated with 10 mM H_2_O_2_. Total *SsSvf1* cDNA abundance in the samples was normalized to using *tub1* gene as a control. The relative expression of *SsTrr1* in the untreated strain was set as 1. Bars indicate standard error. **(B)** Percent growth inhibition of wild-type strain and *SsTrr1* gene-silenced strains on PDA medium with 5 and 10 mM H_2_O_2_. Bars indicate standard deviation. Asterisks denote significant differences (one-way ANOVA). ^∗∗^*P* < 0.01.

The relative *SsTrr1* expression levels under oxidative stress were determined via real-time RT-PCR in order to extensively characterize the role of *SsTrr1* in response to oxidative stress in *S. sclerotiorum*. As shown Figure 8A, the *SsTrr1* expression level increased sharply in hyphae that were treated with 10 mM H_2_O_2_. The hyphal growth under oxidative stress between the wild-type and *SsTrr1* gene-silenced strains was then compared. When growth on PDA plates was amended with H_2_O_2_, hyphal growth inhibition was significantly greater for SiTrr1-54 and SiTrr1-59 than the wild-type strain (Figure 8B). The results indicate that *SsTrr1* contributes to the oxidative stress tolerance in *S. sclerotiorum*.

## Discussion

In this study, the gene *SsTrr1*, which encodes a TrxR in *S. sclerotiorum*, was cloned and functionally analyzed. *SsTrr1* was shown to have an effect on the oxidative stress tolerance, sclerotial development, and pathogenicity of *S. sclerotiorum*.

Evidence has shown that the numbers of TrxRs vary among different fungi. In *S. cerevisiae*, two TrxRs (Trr1 and 2) were identified and were shown to be located in the cytoplasm and mitochondria, respectively (Pearson and Merrill, 1998; Pedrajas et al., 1999). The filamentous fungal insect pathogen *B. bassiana* also contains two TrxRs that play distinct roles in the redox system and host infection (Zhang et al., 2016). However, only one TrxR was identified in fungal plant pathogens, including *B. cinerea*, *M. oryzae*, and *A. alternata*, and a cytoplasmic location was demonstrated in these fungi (Fernandez and Wilson, 2014; Viefhues et al., 2014; Ma et al., 2018). BLASTP searches indicated that *S. sclerotiorum* contains only one TrxR-encoding gene, and this number is consistency with other fungal plant pathogens. Since there is a lack of effective fluorescent protein labeling methods, the subcellular localization of TrxR in *S. sclerotiorum* was predicted using ProtCom 9.0 servers^1^, and the results showed that SsTrr1 is most likely localized in the cytoplasm.

Some genes that are related to ROS modulation in *S. sclerotiorum* have been discussed. Functional loss of a Cu/Zn superoxide dismutase in *S. sclerotiorum* resulted in an increase in sensitivity to oxidative stress in culture (Veluchamy et al., 2012; Xu and Chen, 2013). However, deletion of the catalase, SCAT1, in *S. sclerotiorum* led to an increase in tolerance to H_2_O_2_, indicating that SCAT1 is not essential for H_2_O_2_ degradation *in vitro* (Yarden et al., 2014). In recent years, several fungal TrxR-encoding genes have been shown to play critical roles in oxidative stress responses (Fernandez and Wilson, 2014; Viefhues et al., 2014; Ma et al., 2018). In this study, *SsTrr1* showed a sharp increase in expression under oxidative stress conditions, and the gene-silenced strains exhibited sensitivity to H_2_O_2_, suggesting a conserved function for TrxR in oxidative stress in fungi.

The generation of ROS has been recorded as one of the earliest resistance responses for plants against fungal pathogens (Bolwell et al., 1995). ROS detoxification and tolerance are critical for *S. sclerotiorum* hyphae to infect host plants successfully (Kim et al., 2011; Williams et al., 2011; Yarden et al., 2014). Previous evidence has shown that these genes, which play critical roles in the detoxification and tolerance of ROS, are essential for *S. sclerotiorum* pathogenesis (Veluchamy et al., 2012; Xu and Chen, 2013; Yu et al., 2015). McLoughlin et al. (2018) reported that foliar applications of dsRNA-targeted *SsTrr1* reduced *S. sclerotiorum* infection in *B. napus*. In this study, we found that *SsTrr1* gene-silenced strains exhibited attenuated virulence in different hosts. SiTrr1-59 with a lower expression level of *SsTrr1* led to smaller lesions. These data further indicted that TrxR is critical for the successful infection of this fungus. The role of TrxR in *S. sclerotiorum* virulence is consistent with those of fungal plant pathogens such as *B. cinerea* (Viefhues et al., 2014), *A. alternata* (Ma et al., 2018), and *M. oryzae* (Fernandez and Wilson, 2014), in addition to the fungal insect pathogen *B. bassiana* (Zhang et al., 2016). It is suggested that the absence of a component of the Trx system may lead to a disturbance of fungal redox balance, which is critical for fungal infection and colonization.

Sclerotia are important dormant bodies for many fungal plant pathogens in Ascomycota and *Basidiomycota*, including *S. sclerotiorum*, *B. cinerea*, *Rhizoctonia solani*, and *Verticillium dahliae*. Sclerotial development in *S. sclerotiorum* is a complicated biological process and can be divided into three distinguishable stages: (1) initiation, (2) development, and (3) maturation (Willetts and Bullock, 1992). Sclerotial formation is affected by several environmental signals, including nutrient limitation, light, pH, and temperature, and is under the control of cyclic adenosine monophosphate (cAMP)/protein kinase A (PKA) and mitogen-activated protein kinase (MAPK) cellular signaling pathways (Chet and Henis, 1975; Chen et al., 2004; Jurick et al., 2004; Jurick and Rollins, 2007). Classical theory postulates that hyperoxidant states trigger microbial eukaryotic cell differentiation (Lara-Ortiz et al., 2003; Aguirre et al., 2005). Previous studies have also shown that sclerotial differentiation is associated with an increase in oxidative level, and oxidative stress promoted sclerotial metamorphosis (Georgiou et al., 2006; Papapostolou et al., 2014). Silencing of the NADPH oxidase genes *SsNox1* and *SsNox2* resulted in reduced ROS levels and limited sclerotial development of *S. sclerotiorum* (Kim et al., 2011). However, a Cu/Zn superoxide dismutase gene-deletion mutant exhibited normal sclerotial formation (Xu and Chen, 2013), indicating a complex role for ROS in the sclerotial development of *S. sclerotiorum.*

In this study, sclerotial development was suppressed by auranofin, which inhibited TrxR activity. Furthermore, targeted silencing of the TrxR-encoding gene *SsTrr1* led to the production fewer of sclerotia. To our knowledge, this is the first report that TrxRs are required for fungal sclerotial development. However, the level of *SsTrr1* expression exhibited a decrease when sclerotia began to form, which indicated a dynamic balance of TrxR activity is critical for sclerotial development. Since TrxR is critical and consists of Trx systems that act against oxidative stress, we hypothesized that TrxR impacts sclerotial development via intracellular redox-level regulation. *S. sclerotiorum* often produces a ring of sclerotia at the edge of the colony, mainly because of inhibition of polar elongation, staling compounds, and nutrient limitation (Chet and Henis, 1975; Jurick and Rollins, 2007). In this study, targeted silencing of *SsTrr1* resulted in sclerotial formation in a random manner, and most of the sclerotia were located in the center of the plates. Since *SsTrr1* gene-silenced strains showed normal hyphal growth, *SsTrr1* silencing may lead to impaired sensing of staling compounds and nutrient limitation. It is interesting to note that catalase SCAT1 deletion strains also produced sclerotia in the center of the plates (Yarden et al., 2014), indicating that the maintenance of the redox status is indeed necessary for the sclerotial development of *S. sclerotiorum*. The connection between the substrates of TrxRs and the sclerotial development of *S. sclerotiorum* requires additional studies.

## Author Contributions

JZ, YW, JD, ZH, AF, YhY, CB, LQ, and YY conceived and designed the experiments, and contributed reagents, materials, and analysis tools. JZ and YY performed the experiments, analyzed the data, and wrote the manuscript. All authors read and approved the final manuscript.

## Conflict of Interest Statement

The authors declare that the research was conducted in the absence of any commercial or financial relationships that could be construed as a potential conflict of interest.

## Footnotes

1 http://www.softberry.com/
